# Temporal sampling helps unravel the genetic structure of naturally occurring populations of a phytoparasitic nematode. 2. Separating the relative effects of gene flow and genetic drift

**DOI:** 10.1111/eva.12401

**Published:** 2016-07-22

**Authors:** Cécile Gracianne, Pierre‐Loup Jan, Sylvain Fournet, Eric Olivier, Jean‐François Arnaud, Catherine Porte, Sylvie Bardou‐Valette, Marie‐Christine Denis, Eric J. Petit

**Affiliations:** ^1^ IGEPPINRA, Agrocampus OuestUniversité Rennes 1Le RheuFrance; ^2^ VetAgro Sup, UMR 1095, GDECClermont UniversitéClermont‐FerrandFrance; ^3^ ESE, Ecology and Ecosystems HealthAgrocampus OuestINRARennesFrance; ^4^ UMR CNRS 8198 ÉvolutionÉcologie et PaléontologieUniversité Lille 1 ‐ Sciences et TechnologiesVilleneuve d'Ascq CedexFrance

**Keywords:** assignment tests, gene flow, *Heterodera schachtii*, migration–drift equilibrium, wild nematode populations

## Abstract

Studying wild pathogen populations in natural ecosystems offers the opportunity to better understand the evolutionary dynamics of biotic diseases in crops and to enhance pest control strategies. We used simulations and genetic markers to investigate the spatial and temporal population genetic structure of wild populations of the beet cyst nematode *Heterodera schachtii* on a wild host plant species, the sea beet (*Beta vulgaris* spp. *maritima*), the wild ancestor of cultivated beets. Our analysis of the variation of eight microsatellite loci across four study sites showed that (i) wild *H. schachtii* populations displayed fine‐scaled genetic structure with no evidence of substantial levels of gene flow beyond the scale of the host plant, and comparisons with simulations indicated that (ii) genetic drift substantially affected the residual signals of isolation‐by‐distance processes, leading to departures from migration–drift equilibrium. In contrast to what can be suspected for (crop) field populations, this showed that wild cyst nematodes have very low dispersal capabilities and are strongly disconnected from each other. Our results provide some key elements for designing pest control strategies, such as decreasing passive dispersal events to limit the spread of virulence among field nematode populations.

## 1 Introduction

Agrosystems are highly homogenous artificial environments that are particularly amenable to the emergence and development of pathogens (Stukenbrock & McDonald, 2008). In such artificial, disturbed habitats, gene flow is a crucial parameter that determines the adaptive value of pathogen populations and the risk they represent for crops, especially through the evolution of virulence and resistance breakdown (McDonald & Linde, 2002). This is particularly true for pathogens with high dispersal abilities that have alternative wild hosts outside the cropping area even in the absence of crops (Burdon & Thrall, 2008). There is increasing evidence that crop pathogens can develop on wild host species, related or unrelated to the usual cultivated host (e.g. Lebeda, Petrželová, & Maryška, 2008; Monteil et al., 2013; Rouxel et al., 2014). Therefore, wild populations of pathogens may act as reservoirs of genetic diversity and initiate local crop epidemics (Burdon & Thrall, 2008; Leroy, Le Cam, & Lemaire, 2014). In this respect, a few studies on wild plant pathogen populations have investigated patterns of gene flow between wild and cultivated hosts (see examples in Stukenbrock & McDonald, 2008). However, there are no published reports on the patterns of gene flow among wild plant pathogen populations, which are nonetheless an important determinant of plant pathogen population structure and thus of its potential role as a virulence reservoir.

Studying wild plant pathogen populations offers the opportunity to better understand the evolutionary dynamics of crop pathogen populations and can provide clues on the influence of human activities on the genetic structure of pathogen populations in agrosystems (Lebarbenchon, Brown, Poulin, Gauthier‐Clerc, & Thomas, 2008; Morgan, Clare, Jefferies, & Stevens, 2012). Although advocated, this approach has received very little attention thus far, with only one study on readily identifiable zoopathogens (see Morrison & Hoglund, 2005). In the case of soilborne plant diseases, there is no information about wild populations of pathogens, probably because they are difficult to diagnose. Among soilborne pathogens, plant‐parasitic nematodes are major crop pests of agrosystems that can cause severe economic losses annually (Jones et al., 2013). However, there are few detailed investigations of their population genetic structure, and even scarcer information on their spatial genetic structure in the wild (Gilabert & Wasmuth, 2013; van der Putten et al., 2006). All available data come from two studies on the pinewood nematode, *Bursaphelenchus xylophilus*, and four crop field surveys on the dagger nematode *Xiphinema index* and three cyst nematodes *Globodera pallida*,* Globodera tabacum*, and *Heterodera schachtii* (Alenda, Montarry, & Grenier, 2014; Mallez et al., 2013, 2015; Picard & Plantard, 2006; Plantard & Porte, 2004; Villate, Esmenjaud, Van Helden, Stoeckel, & Plantard, 2010). The latter three plant‐parasitic nematodes cause severe damage to vineyards and potato fields in Europe, tobacco fields and sugar beet fields worldwide, respectively. *Bursaphelenchus xylophilus* damages pine forests around the world and differs from cyst nematode species in its life history, requiring an intermediary insect species to disperse from one pine tree to another. The population genetic structure of this nematode indicates that *B. xylophilus* populations can be differentiated among individual trees at a very small spatial scale, according to the dispersal of the insect vector (Mallez et al., 2013). In contrast, cyst nematodes are soilborne endoparasitic nematodes with direct life history cycles, in which only two free‐living developmental stages (males and juveniles) can disperse actively in the soil. Due to their small size (<1 mm), nematodes can travel over short distances only (Norton & Niblack, 1991; Wallace, 1968). Interestingly, populations of *G. pallida* and *H. schachtii* exhibit low genetic differentiation among cultivated fields located 50 and 150 km apart, respectively, suggesting the occurrence of gene flow over large spatial scales. This pattern was attributed to large nematode population sizes in cultivated fields and passive dispersal of cysts in agricultural areas via natural factors and human activities (Alenda et al., 2014; Picard & Plantard, 2006; Villate et al., 2010). However, there have been no investigations on wild populations of crop plant‐parasitic nematodes to date, and the question remains as to whether plant‐parasitic nematodes also passively disperse over large distances in wild ecosystems.

In this study, we examined the genetic structure of wild populations of a cyst nematode, *H. schachtii*, using both empirical and simulated datasets. Sugar beet (*Beta vulgaris* spp. *vulgaris*) is the usual cultivated host of *H. schachtii* which can also develop on wild relative of the sugar beet, the sea beet *B. vulgaris* spp. *maritima* (Subbotin, Mundo‐Ocampo, & Baldwin, 2010). Sea beets are common along the European Atlantic and Mediterranean coastlines (De Cauwer, Dufay, Cuguen, & Arnaud, 2010; Hautekèete, Piquot, & Van Dijk, 2002). Wild populations of *H. schachtii* have been found on wild sea beet populations located along the Atlantic coastline from Spain to Denmark (Gracianne et al., 2014). This wide geographical occurrence provides the opportunity to explore the patterns of gene flow over different spatial scales in the wild. Specifically, (i) we investigated the levels of genetic differentiation in local wild populations of *H. schachtii* to examine what hierarchical scale should be considered to define a population in the wild, (ii) we examined the spatial genetic structure of nematode populations to identify signatures of dispersal events and the scale over which they occur, and (iii) we separated the respective effects of gene flow and genetic drift in observed population genetic structure.

## 2 Materials and Methods

### 2.1 Biological material and sampling design

Soil samples were collected around roots of *B. vulgaris* spp. *maritima* plants on four different beaches in Normandy, France. Distances separating the four beaches (Montfarville, Granville Nord, Granville Sud, and Saint Léonard) ranged from 300 m to 150 km (for more details, see Jan et al., 2016) to mimic the geographical sampling scheme that was performed in a previous investigation on *H. schachtii* conducted in sugar beet fields (Plantard & Porte, 2004). Populations of the host plant, the sea beet, are often composed of individuals clustered in geographically and genetically distinct patches (De Cauwer et al., 2010). Thus, a maximum of 10 sea beet plants were sampled in three to five arbitrarily defined patches of 3 m in diameter randomly distributed on each beach (Fig. S1). Those patches do not have any biological meanings related to the nematode biology and were only used for delineating sampling areas. In fall 2012, 120 plants were sampled and marked with plastic tags. Due to their small size (<1 mm), active dispersal of *H. schachtii* individuals occurs over very short spatial distances (Plantard & Porte, 2004; Wallace, 1968; Westphal, 2013). Thus, the soil sampled around the roots of one plant was considered as being one nematode population for subsequent analyses. Thousands of cysts were found in all soil samples. To reduce the amount of biological material, molecular analyses were performed, for each beach, on all nematode populations coming from one sea beet patch and on one nematode population coming from the other patches of sea beets (Fig. S1). We finally genotyped 40 nematode populations sampled in 2012. In fall 2013, only 34 of those 40 populations were resampled because six host plants disappeared between the two sampling dates. Cysts of *H. schachtii* were extracted from soil samples using homemade sieves (250 and 800 μm) and manual examination of filtrates. Cysts were stored at 4°C in moistened sand until molecular characterization.

### 2.2 Molecular characterization and genotyping

As a soil sample can contain cysts from different *Heterodera* species, we used restriction profiles of the ITS sequence for species identification. DNA extraction, PCR amplification of ITS sequence and digestion of PCR products were performed as described in Amiri, Subbotin, and Moens (2002). Polymorphic mitochondrial markers are not yet described in *H. schachtii*, but we used nuclear microsatellite loci that are markers of choice for studying neutral genetic structure. In all, 1,754 *H. schachtii* individuals were identified and successfully genotyped at eight microsatellite loci, named Hs33, Hs36, Hs55, Hs56, Hs68, Hs84, Hs111, and Hs114 and described in Montarry et al. (2015). Microsatellites PCR products were analyzed on an ABI Prism^®^ 3130xl sequencer (Applied Biosystems, Foster City, CA, USA). Allele sizes were identified using the automatic calling and binning procedure of GeneMapper v4.1 (Applied Biosystems) and completed by a manual examination of irregular results. Samples with dubious genotypes were reamplified.

### 2.3 Data Analyses

#### 2.3.1 Dataset and population partitioning

In our sampling scheme, populations of *H. schachtii* were a priori defined at the scale of a single host plant because active dispersal of *H. schachtii* is considered to be spatially restricted and because it is very difficult to perform fine‐scaled spatial sampling over the root system of the host plant. However, we assessed the scale of population boundaries using three different analyses. We first used a spatial principal component analysis (sPCA) to investigate the spatial distribution of genetic diversity within each surveyed beach using the R package adegenet (Jombart, 2008). This method is not based on any population genetic hypothesis and summarizes the genetic variation into synthetic variables maximizing the product of the variance taking into account the spatial autocorrelation between sampling locations using Moran's *I*, which was calculated using a network based on Delaunay triangulation. Synthetic components can be positive or negative reflecting, respectively, global or local structure (i.e. positive or negative spatial autocorrelation among genetic units). The two‐first principal component scores were simultaneously represented into a channel of color to draw a comprehensive synthetic representation of sPCA scores, as described in Menozzi, Piazza, and Cavalli‐Sforza (1978). We also used pairwise kinship coefficients *F*_*ij*_ among nematode individuals to identify the scale over which a spatial genetic structure may appear among host plants (Loiselle, Sork, Nason, & Graham, 1995). This analysis describes kinship variation among individuals over spatial distances which can indicate the scale over which a significant spatial genetic structure occurs. Standard errors of *F*_*ij*_ were estimated using a jackknifing procedure among loci implemented in the software SPAGeDi version 1.5 (Hardy & Vekemans, 2002). We defined eight to eleven geographical distances classes for each dataset considering an even distribution of pairwise individuals and computed 95% confidence intervals using 10,000 permutations of individual locations to test whether average kinship coefficients significantly departed from zero. The spatial scale of positive autocorrelation, defining genetic neighborhood in the broad sense, was considered as the distance value for which *F*_*ij*_ coefficients dropped under zero (see Favre‐Bac, Mony, Ernoult, Burel, & Arnaud, 2016; Sokal & Wartenberg, 1983). Finally, we performed a nonspatially explicit Bayesian genetic clustering on the 34 populations sampled in 2012 and in 2013 to detect potential substructuring both at the beach and at the host plant scale, using STRUCTURE (Pritchard, Stephens, & Donnelly, 2000). Each *K* value, ranging from 1 to 25, was tested with 30 replicated runs consisting in a burn‐in period of 100,000 iterations followed by 2.10^6^ Monte Carlo Markov Chain replications.

Results allowed us to define populations of *H. schachtii* at the host‐spatial scale (see below) in all the subsequent analyses. Analyses were performed on the whole 2012 dataset (*n* = 40 populations), and 34 of these populations were resampled in 2013. Jan et al. (2016) identified 14 populations that were not able to produce enough generations to detect substantial genetic variation between the two sampling sessions. Because it is not clear what effects these populations could have on our results, we analyzed two different datasets for 2013: one dataset with all sampled populations (*n* = 34) and one dataset that was restricted to populations that produced enough generations between 2012 and 2013 to detect substantial temporal genetic variation (Table 1). Both kinds of analyses led to the same conclusions. In the following, we will present results based on the restricted 2013 dataset (*n* = 20) and will include results from the unrestricted dataset (*n* = 34) when they provide useful additional information.

**Table 1:** Genetic diversity and summary statistics for nematode populations sampled in 2012 and 2013

Site	Plant code	*n*		*A*_r_		*H*_e_		*F*_IS_	
		2012	2013	2012	2013	2012	2013	2012	2013
Granville Nord	Fra.71N.P1.4	25	18	2.09	2.04	0.409	0.390	.085	.124
	Fra.71N.P2.1	25	23	1.78	1.96	0.324	0.361	−.162^a^	.143^a^
	Fra.71N.P2.2	25	16	1.93	1.94	0.361	0.346	.056	.284^a^
	*Fra.71N.P2.3*	25	30	2.06	2.06	0.390	0.384	.021	.094
	*Fra.71N.P2.4*	23	27	1.90	2.07	0.358	0.396	.021	.137^a^
	Fra.71N.P2.5	22	27	2.08	2.04	0.412	0.380	−.04	.189^a^
	Fra.71N.P2.6	24	–	1.87	–	0.379	–	.013	–
	Fra.71N.P2.7	24	–	1.91	–	0.344	–	.123	–
	Fra.71N.P2.8	22	35	2.07	2.05	0.371	0.380	.071	.151^a^
	*Fra.71N.P3.7*	21	35	2.01	2.00	0.398	0.388	.051	.192^a^
	*Fra.71N.P4.1*	27	38	1.89	1.83	0.331	0.306	.103	.170^a^
	*Fra.71N.P5.2*	26	32	1.92	1.93	0.361	0.361	.162^a^	.171^a^
Granville Sud	Fra.7_1.P2.5	24	–	1.90	–	0.345	–	−.072	–
	Fra.7_1.P3.7	19	19	2.02	2.02	0.421	0.415	.144^a^	.129
	*Fra.7_1.P4.1*	20	22	1.85	1.89	0.339	0.333	.136	.185^a^
	Fra.7_1.P5.1	22	25	2.29	2.06	0.445	0.407	.101	.222^a^
	*Fra.7_1.P5.2*	25	25	1.94	1.86	0.341	0.312	.145^a^	.092
	Fra.7_1.P5.3	25	32	1.79	1.80	0.294	0.312	.030	−.076
	*Fra.7_1.P5.4*	20	30	1.91	1.93	0.344	0.355	.014	.191^a^
	Fra.7_1.P5.6	20	23	2.11	2.05	0.381	0.374	.036	.281^a^
	*Fra.7_1.P5.7*	25	32	2.22	2.08	0.418	0.376	.008	.099^a^
	*Fra.7_1.P5.8*	13	37	2.01	2.08	0.349	0.367	.080	.041
	Fra.7_1.P5.10	23	–	2.09	–	0.365	–	.108	–
Saint Léonard	Fra.7_4.P1.1	21	25	2.06	1.82	0.398	0.316	−.243^a^	.201^a^
	Fra.7_4.P1.2	24	19	1.86	1.89	0.325	0.344	−.054	.362^a^
	Fra.7_4.P1.3	13	11	1.87	1.73	0.335	0.276	.025	.141
	Fra.7_4.P1.7	24	22	1.82	1.91	0.292	0.365	−.218^a^	−.077
	Fra.7_4.P1.8	24	17	1.84	1.70	0.339	0.293	.096	.125
	*Fra.7_4.P1.9*	19	28	1.97	1.87	0.368	0.328	.070	.058
	Fra.7_4.P1.10	18	–	1.91	–	0.352	–	.015	–
	Fra.7_4.P2.1	29	17	1.86	1.71	0.318	0.296	.063	.138
	*Fra.7_4.P3.5*	12	17	1.53	1.43	0.188	0.172	−.140	−.112
Montfarville	*Fra.8_4.P1.1*	25	21	1.96	1.92	0.354	0.339	.391^a^	.199^a^
	Fra.8_4.P1.2	25	22	2.10	1.87	0.377	0.314	.269^a^	.294^a^
	Fra.8_4.P1.3	25	24	2.12	1.98	0.408	0.351	−.307^a^	.128^a^
	Fra.8_4.P1.4	22	33	2.00	1.92	0.343	0.315	.192^a^	.235^a^
	Fra.8_4.P1.5	28	26	2.14	2.04	0.385	0.371	.097	.262^a^
	Fra.8_4.P2.7	17	24	2.04	1.96	0.375	0.374	.211^a^	.205^a^
	*Fra.8_4.P2.9*	23	22	2.08	2.12	0.345	0.368	.165^a^	.360^a^
	Fra.8_4.P3.1	26	–	2.17	–	0.409	–	−.094	–

Italicized population names show populations with infinite effective population sizes. *n*, number of genotypes; *A*_r_, allelic richness; *H*_e_, expected heterozygosity.

a indicates significant *F*_IS_.

#### 2.3.2 Characterization of basic genetic parameters

Hardy–Weinberg (HW) equilibrium is required in population assignment‐based methods (see below). Therefore, single and multilocus departures from HW equilibrium were tested by estimating *F*_IS_ values for all populations on each beach. Statistical significance of *F*_IS_ values was assessed using 10,000 permutations of alleles among individuals, adjusted for multiple tests with Bonferroni corrections, as implemented in the software FSTAT version 2.9.3 (Goudet, 1995). Similarly, linkage disequilibrium among loci was also assessed using permutation tests adjusted with Bonferroni corrections implemented in FSTAT. Genetic diversity of nematode populations was evaluated through the estimation of expected heterozygosity (*H*_e_) and allelic richness (*A*_r_) using FSTAT. Allelic richness was estimated using the rarefaction method as described in El Mousadik and Petit (1996).

#### 2.3.3 Levels of genetic differentiation

Three hierarchical levels of population structure were considered to explain the partitioning of genetic differentiation: the level of the host plant, considered as hosting one nematode population, as described above; the level of the patch, defined as a geographical clustering of host plants within a beach; and the level of the beach, comprising all the set of host plants surveyed for nematode population sampling. We used the R package hierfstat (Goudet, 2005) to estimate variance components of each hierarchical level and to test their statistical significance, as described in De Meeûs and Goudet (2007). To test for isolation by distance (IBD), that is a gradual increase of genetic differentiation with increasing geographical distance among populations, we estimated pairwise population *F*_ST_ and performed regression analyses as described in Rousset (1997) using the R package adegenet (Jombart, 2008). Significance of the relationship between the two variables was tested using classical Mantel tests (Smouse, Long, & Sokal, 1986). The same analyses were performed on both (2012 and 2013) datasets.

#### 2.3.4 Migrant detection: empirical data

At migration–drift equilibrium, a pattern of IBD depicted through variation in genetic differentiation among populations reflects spatially restricted gene flow. However, such population structure can be a residual signal of ancestral gene flow that no longer occurs or can be the result of diverse nonequilibrium situations such as colonization (Barker, 2013), secondary contact between allopatric populations (Petrou et al., 2013), or population expansion (Awad, Fady, Khater, Roig, & Cheddadi, 2014). Thus, to help us distinguish equilibrium from nonequilibrium situations in IBD patterns, we used assignment tests to detect real‐time (i.e. first‐generation) migrants, which are assumed to reflect current gene flow (Broquet & Petit, 2009; Manel, Gaggiotti, & Waples, 2005). The detection of first‐generation migrants and population assignment of individuals were conducted using Bayesian criteria based on the computation of the probability of observing a given genotype in each population (see Rannala & Mountain, 1997 for details). This approach is generally used when all population sources of the detected first‐generation migrants are not known. Using GeneClass 2 (Piry et al., 2004), this approach was applied to define the statistical criteria that estimate the likelihood that an individual originates from a given population. The detection of first‐generation migrants was performed by computing the likelihood of the individual genotype within the population where the individual was sampled. For each individual, the probabilities of belonging to each sampled population were estimated by simulating 10,000 multilocus genotypes using a Monte Carlo resampling procedure, as described in Paetkau, Slade, Burden, and Estoup (2004). Individuals with a probability lower than .01 of occurring in the sampled population were considered as potential migrants. This threshold corresponds to the minimal tolerable type I error expected from assignment tests using this procedure (Paetkau et al., 2004). Assignments of individuals to a population were based on computation of individual exclusion probabilities. In this case, the population exhibiting the highest membership probability was considered as the population of origin of the individual. Individuals assigned to another population from which they were sampled were considered as migrants. Migrant detection was thus probabilistic and did not aim at identifying the origin of migrants, because it is not possible to sample all populations in our system (Paetkau et al., 2004; Piry et al., 2004). Migrant origins could be diverse, with different effects on IBD. Migrants that originate from populations located nearby sampled populations (short‐distance dispersal) could contribute to maintain IBD. Migrants that originate from populations situated outside the sampled beaches (long‐distance dispersal) or from encysted stages from the sampled populations (temporal dispersal) would not contribute to maintain IBD but could more likely disrupt it.

Assignment tests assumed HW equilibrium. In 2012, one locus in Saint Léonard, two loci in Montfarville and in Granville Nord, and three loci in Granville Sud exhibited significant departure from HWE (see below). In 2013, three loci in Montfarville and Granville Sud, four loci in Saint Léonard, and seven loci in Granville Nord showed significant departures from HWE. To assess the influence of loci departing from HWE on results, assignment tests were performed with and without these loci for populations sampled in 2012. Results did not show any difference in the distribution of detected migrants over distance among populations (data not shown). As a result, we considered results from assignment tests considering all loci in 2012 and 2013 in subsequent analyses. All analyses computed on empirical datasets were performed separately for the different beaches.

#### 2.3.5 Migrant detection: simulations

Wang (2014) recently showed that assignment‐based methods can greatly overestimate migration rates in IBD contexts. We thus decided to evaluate the rate of false‐positive migrants that assignment tests may generate in our analyses by simulating datasets that mirror our empirical population genetic data. To do so, we simulated a grid of 9 × 9 (81) populations with balanced sex ratio using the software EasyPop version 2.0.1 (Balloux, 2001). This grid is a very simplified representation of the spatial structure of nematode populations, but we chose to use it because (i) we did not sample all sea beets within patches, and thus all *H. schachtii* populations, and (ii) because we did not have any representation of the root systems which may bring populations closer or farther apart than what could be expected from the spatial distribution of plants. In this case, a square was the simplest experimental design to investigate dispersal occurring among the closest populations, that is among adjacent populations whatever the geographical distance among them. Assuming random mating within each simulated population, we used a two‐dimensional stepping‐stone migration model because it reflects the spatial positions of host sea beet plants, and it entails that migration events only occur among adjacent host plants, in accordance with the restricted dispersal capabilities in *H. schachtii*. We explored four different scenarios by varying effective sizes and migration rates. Effective sizes *N*_e_ were set at 100 and 400 individuals, and migration rates *m* at 0.04 and 0.1, and 0.1 and 0.025, respectively, to explore the behavior of assignment tests in situations that correspond to *N*_e_ *m* = 4 and *N*_e_ *m* = 10, which are the two most extreme values for the product *N*_e_ *m* we observed in the four beaches. The mutation rate was fixed at 5 × 10^−4^ mutation/locus/generation (Molnar, Witte, Dinkelacker, Villate, & Sommer, 2012; Seyfert et al., 2008) and followed a K‐allele model with eight possible allelic states, which corresponded to the maximal number of alleles observed in our microsatellite loci dataset. We chose to simulate only five independent loci to mimic worst conditions in empirical datasets (i.e. the smallest number of loci at HW equilibrium in 2012). Each simulation spanned enough time to reach migration–drift equilibrium (2000 generations). We randomly sampled 20 individuals in 3 × 3 simulated populations, the same number as in the empirical dataset, from each of the 100 replicates of the simulation procedure. Sampled populations were contiguous and formed a square that was located in the center of the simulated grid to avoid potential border effects. Under a strict stepping‐stone model of population structure, we expected to detect migrants between adjacent populations only, and none for all other increasing distance classes, other than type I errors. Statistical detection of migrants was performed as for empirical data. Handling and analysis of all datasets were performed using R version 3.1.1 (R Development Core Team 2012). The percentage of false positives was computed as the proportion of migrants that were detected among nonadjacent populations over the total number of detected migrants overall replicates for each simulation scenario.

#### 2.3.6 Impact of genetic drift

According to the results presented in Jan et al. (2016), *H. schachtii* populations have low effective population sizes at the host plant scale with a mode at *N*_e_ = 85. Genetic drift may thus have a substantial impact on the population genetic structure in this nematode. Actually, in the case of no or low gene flow, genetic drift would be the only evolutionary force that may significantly affect variation in allelic frequencies between samples taken a few generations apart (a 1‐year sampling interval corresponds to a maximum of 10 generations in wild *H. schachtii* populations, see Jan et al., 2016). Natural selection is expected to have negligible effect on the variation of neutral markers such as microsatellites, and the expected number of mutants over ten generations is less than one per locus (10 × 5.10^−4^ × 85 = 0.42), given the mutation rate observed in nematodes (Molnar et al., 2012; Seyfert et al., 2008).

At migration–drift equilibrium, the slope of regression line of IBD patterns reflects the influence of both gene flow and genetic drift. If genetic drift is the only evolutionary force that acts on population structure, the slope of IBD patterns should progressively decrease due to the increase in genetic differentiation among populations. Similarly, mean and variance of *F*_ST_ should increase over time. If such variation is indeed observed in our data, observed IBD patterns will have not been generated by current gene flow, but will actually correspond to a residual signal of gene flow that no longer occurs or to another unknown nonequilibrium process.

However, because drift effects accumulate with time and because our sampling period covered between four and ten nematode generations only (see Jan et al., 2016), these variations may not be detectable. To prevent this problem, we simulated the evolution of allele frequencies observed in 2012 under four to ten generations under a model of pure genetic drift. We then compared the resulting population genetic structure with what we observed in 2013. The whole procedure was repeated 1,000 times for each tested *N*_e_ value (10 ≤ *N*_e_ ≤ 2000) to generate distributions of IBD slopes, means, and variances of pairwise *F*_ST_ values that were compared with what we observed in 2013. All simulations, data handling, and testing were performed with R.

## 3 Results

### 3.1 Population definition

Spatial principal component analysis analyses performed over the four surveyed beaches and the two sampling years failed to show a global pattern but showed significant local structure, that is repulsive structure with negative spatial autocorrelation, suggesting that nematodes located on different plants are genetically distinct (Fig. S2). In the same way, spatial genetic structure was only detected among individuals sharing the same host plant in Granville Nord and Montfarville in 2012 and 2013, and up to 80 or 55 cm in Granville Sud (in 2012) and in Saint Léonard (in 2012 and 2013), respectively (see Fig. S2). Beyond this level, average kinship estimates did not depart from spatial randomness. These results suggested a very short‐distance spatial autocorrelation with a lack of detectable IBD within the four studied beaches for both sampling years. For 82% of the 68 tested datasets using Bayesian genetic clustering, *∆K* were <10 which is uninformative and prevent reliable assignment of individuals. In the resting 12%, *∆K* were higher, but in all cases, the most probable number of genetic clusters was 1. This analysis was thus not able to detect any genetic structuring at a beach scale but also at the host plant scale and results were not included in this study.

### 3.2 Genetic data

No significant linkage disequilibrium was detected, and microsatellite loci were consequently considered as independent. Multilocus genotypic data showed contrasting levels of genetic diversity among sampled beaches (Table 1). Forty‐one alleles were observed (ranging from 1 to 7 per locus) and expected heterozygosity (*H*_e_) ranged from 0 to 0.582. The overall average *F*_IS_ was significant in 2012 (*F*_IS_ = 0.041) and in 2013 (*F*_IS_ = 0.153). Some loci showed significant departures from HW equilibrium, which represented 12% and 15% of the 320 and 272 single‐locus tests performed among loci over all populations in 2012 and 2013, respectively. Among these tests, 8% and 15% (4% and 0%) of *F*_IS_ values were significant (positive or negative) in 2012 and 2013, respectively. These values were not associated with a particular locus or population, suggesting spurious effects of genetic drift and/or population genetic substructuring. As a result, 30% and 65% of populations exhibited significant multilocus *F*_IS_ values ranging from −0.307 to 0.391 and from 0.099 to 0.362 in 2012 and 2013, respectively (Table 1). Some (20% in 2012 and 65% in 2013) were significantly positive, suggesting heterozygote deficiencies (Table 1).

### 3.3 Population differentiation and IBD

Only 1.5% and 1.8% of the genetic variance could be attributed to differences between host plant patches in 2012 and 2013, respectively. Population differentiation explained by host plant patch membership was low, or virtually absent, and marginally significant only for the 2012 dataset. In contrast, populations were significantly different among beaches and host plants in 2012 (*F*_Beaches/Total_ = .120; *F*_Plants/Beaches_ = .049; all at *p* < .001) and in 2013 (*F*_Beaches/Total_ = .146; *F*_Plants/Beaches_ = .051; all at *p* = .001).

**Figure 1:**
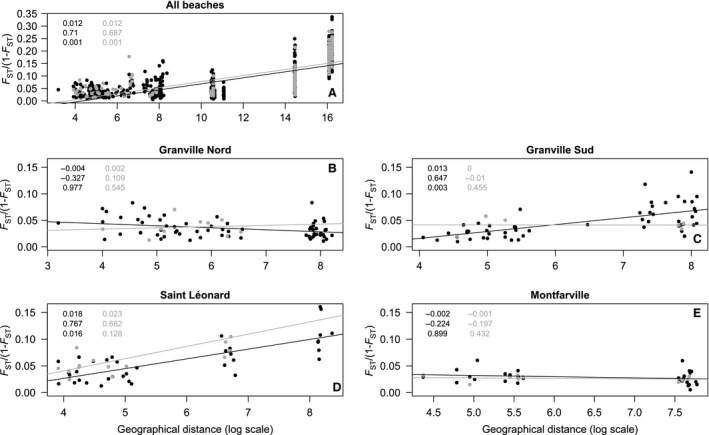
Patterns of isolation by distance (IBD). Black and gray dots and lines correspond to 2012 and 2013 data, respectively. Displayed scores correspond, in descending order, to slopes of the regression line, the *rz* Mantel correlation coefficient, and *p*‐value of Mantel tests for 2012 (black) and 2013 (gray). Regression lines of genetic differentiation with respect to distance (log scale) for all populations on all beaches (A), for populations at Granville Nord (B), for populations at Granville Sud (C); populations at Saint Léonard (D), for populations at Montfarville (E)

There was a significant IBD signature over the whole dataset (*p* < .01 in 2012 and 2013; Fig. 1A). Within single beaches, we observed contrasting situations with either nonsignificant (Granville Nord and Montfarville for both sampling sessions; see Fig. 1B, E) or significant IBD patterns (Granville Sud and Saint Léonard for populations sampled in 2012 only; see Fig. 1C, D). Analyzing the 34 populations sampled in 2013 gave similar results, except for Granville Sud and Saint Leonard where IBD patterns were also significant in 2013 (data not shown), which suggests that the nonsignificant patterns observed with the restricted dataset may be due to a lack of statistical power.

### 3.4 Detection of migrants

We detected 11 potential migrants in 2012 and 1 in 2013 over the whole empirical dataset. We obtained similar results considering the 34 populations sampled in 2013 with 3 migrants detected over the four beaches. Simulations showed that the type I error rate (here the proportion of false positives in migrant detection) largely exceeded 5%. It was 48% and 59% for *N*_e_ = 100, when *N*_e_ *m* = 4 and *N*_e_ *m* = 10, respectively, and 57% and 66% for *N*_e_ = 400, when *N*_e_ *m* = 4 and *N*_e_ *m* = 10, respectively. Taking these false‐positive rates into account led to a maximum of four individuals that may be considered as true migrants over the four beaches and the 2 years (two in Granville Sud and one in Saint Leonard and in Granville Nord, all in 2012).

### 3.5 Genetic drift

**Figure 2:**
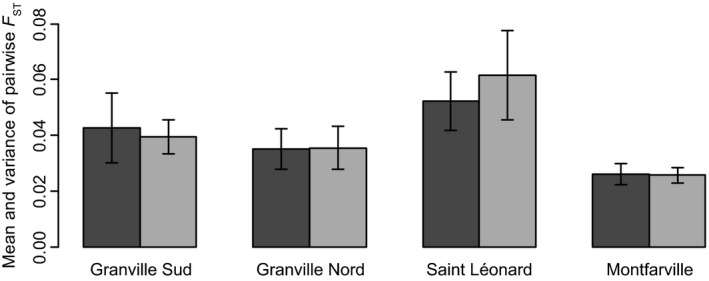
Temporal change in mean pairwise *F*_ST_ values. Black and gray histograms show mean pairwise *F*_ST_ in 2012 and 2013, respectively. Error bars show the standard deviation of pairwise *F*_ST_ values in each beach and sampling year

**Figure 3:**
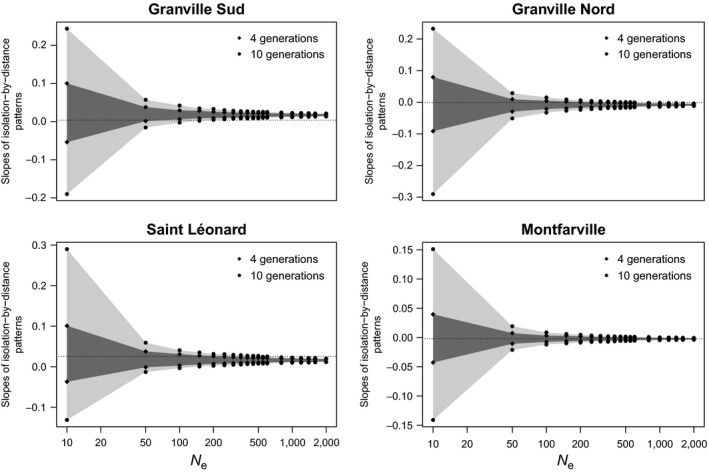
Temporal change in slopes of isolation‐by‐distance (IBD) patterns under a pure drift process. Dark and light gray shapes represent the 95% confidence interval of simulated values obtained after 4 and 10 generations, respectively. Dotted lines indicate the observed slope of IBD patterns in 2013. *N*_e_, effective population size. The *x*‐axis is on a log scale

Isolation by distance patterns differed among sampled beaches, but they remained quite stable between 2012 and 2013 (Fig. 1). Similarly, mean and variance of pairwise *F*_ST_ values did not change between the two sampling sessions (Fig. 2). Simulations showed that the slopes of IBD patterns observed in 2013 were in the 95% confidence interval of what could be expected under pure drift, after four or ten generations, in all beaches (Fig. 3). This result was independent of effective population sizes except for Granville Sud, where the slope of IBD observed in 2013 is laying outside the 95% confidence interval for populations that are larger than 150 individuals. Likewise, the mean and variance of pairwise *F*_ST_ values observed in Granville Nord and Saint Léonard sampled in 2013 were in the 95% confidence intervals of simulated distributions of those variables, except for effective sizes lower than 50 individuals (Fig. S3). A similar pattern was observed in Montfarville with mean and variance of pairwise *F*_ST_ that fall outside the 95% confidence interval for *N*_e_ ≤ 100 individuals. Only Granville Sud showed a mean and variance *F*_ST_ value that lay outside the lower confidence interval for all effective sizes (Fig. S3), suggesting that genetic drift was not the only evolutionary process acting on population genetic structure on this beach.

We got similar results considering all the populations sampled in 2013, except for Saint Leonard. In this beach, the observed slope of IBD pattern and mean *F*_*ST*_ in 2013 laid out the 95% confidence interval for *N*_e_ > 500 individuals (data not shown). Overall, simulations of the evolution of allelic frequencies under pure drift are consistent with observed patterns of population genetic structure, at least for effective population sizes that are in the range of what has been estimated for these populations (Jan et al., 2016).

## 4 Discussion

Investigating gene flow among wild populations of a crop pathogen aims at understanding how allelic frequencies evolve within populations in absence of any disturbances caused by human activities. Temporal sampling at the host plant scale in several beaches allowed us to investigate the occurrence and stability of gene flow occurring among populations of the cyst nematode *H. schachtii*. Results showed that levels of gene flow are low, or even absent, among wild populations on short time scale owing to spatially restricted dispersal events of cysts and/or larvae, in contrast to what has been suggested for agrosystems (see Plantard & Porte, 2004).

### 4.1 IBD patterns: the question of migration–drift equilibrium

The genetic differentiation among wild *H. schachtii* populations showed contrasting patterns of population structure among beaches, sampled years, and spatial scales. IBD patterns that are unstable across spatial scales suggest that the population structure is not at migration–drift equilibrium in *H. schachtii* (Hutchison & Templeton, 1999). Therefore, observed IBD patterns may not result from current gene flow.

This hypothesis is consistent with spatial autocorrelation results, which did not support the occurrence of IBD process within the four beaches. This hypothesis is also well in line with our results on first‐generation migrant detection and simulations which showed that most of the (few) migrants detected in our empirical dataset were probably false positives. It is also worth mentioning that even if they are true migrants, they may not all contribute to maintain IBD, depending on their origin (see section “Migrant detection: empirical data”). This small number of detected migrants may be related to the low genetic variability of the microsatellite markers used, which is known to limit the statistical power of assignment tests (Waples & Gaggiotti, 2006). However, the concordance of results from observed population genetic differentiation with simulated data under pure genetic drift suggests that the low observed number of migrant was not just an artifact. According to our results, there were no or, at most, four migrants observed in sampled wild *H. schachtii* populations, which is not enough to maintain the IBD patterns observed in Granville Sud and Saint Léonard. One may argue that only a few migrants are required to prevent population differentiation and counteract the influence of genetic drift (Slatkin, 1985). However, simulated data under genetic drift provided further support that genetic drift alone can explain the observed variation in population genetic structure between the two sampling years for at least three of the four surveyed beaches. This result would not be expected with ongoing gene flow (Hutchison & Templeton, 1999). Our results thus suggest that wild *H. schachtii* populations were not at migration–drift equilibrium and probably exchange few or no genes at scales ranging from 10 cm to 150 km.

In nonequilibrium situations, patterns of genetic structure are dominated by historical factors, such as colonization history (Austerlitz, Mariette, Machon, Gouyon, & Godelle, 2000; Ibrahim, Nichols, & Hewitt, 1996). This may even be more pronounced in populations that are founded by seeds (plants) or cysts (nematodes), which are forms that can survive belowground until local conditions allow their development. Once a potential host plant grows in a place where cysts are present, the local multiplication of nematodes may generate a patchy distribution of populations and heterogeneity in infestation levels among hosts, as observed in several nematode species (Gavassoni, Tylka, & Munkvold, 2001; Jan et al., 2016; Villate et al., 2008). Most nematode movements in coastal populations may be thus related to exceptional events such as high tides during storms. This hypothesis may explain the results observed in the fourth beach, Granville Sud, where it seems unlikely that the loss of the IBD pattern between 2012 and 2013 results from regular gene flow, because we did not detect more migrants on this beach than on the other beaches and because the slope of IBD pattern observed in 2013 in this beach is smaller than expected under pure genetic drift. However, further studies with an exhaustive sampling of all host plants of a beach conducted over several years are required to gain further insight into the influence of colonization processes on the observed patterns of genetic structure.

### 4.2 Definition of wild *H. schachtii* populations

Our results suggest that passive dispersal through cysts movement is actually limited in the wild. This is consistent with the small effective population sizes (*N*_e_ ranging from 50 to 400) observed in wild *H. schachtii* populations (Jan et al., 2016). Small effective sizes are one consequence of limited gene flow among populations and support the hypothesis that *H. schachtii* is a species constituted of genetically disconnected populations in the wild, even at very small spatial scale. Interestingly, a recent study documented a Wahlund effect in some wild *H. schachtii* populations sampled at the host scale, suggesting that active dispersal of this species can also be extremely limited even between subpopulations that occupy the same host plant (Montarry et al., 2015). These subpopulations may thus correspond to the smallest genetic unit at which individuals interbreed, that is the actual boundaries of populations in *H. schachtii* (Waples & Gaggiotti, 2006).

Strong population genetic structure has already been observed in other parasites that have limited or no active dispersal abilities (Blouin, Liu, & Berry, 1999). Actually, small isolated populations are usually expected in parasites (Price, 1980), particularly in wild plant pathosystems characterized by low host densities, but was barely observed in plant parasite species. In these pathosystems, genetic drift likely has a strong influence and wild parasite populations are more vulnerable to local extinction and/or are maladapted to their local hosts due to their lower dispersal capabilities compared with that of their hosts (Gandon, Capowiez, Dubois, Michalakis, & Olivieri, 1996; Morgan, Gandon, & Buckling, 2005; Thrall & Burdon, 2002). However, small effective population sizes and low levels of gene flow are far from typical in pathosystems, especially when passive dispersal through wind (e.g. Fournier & Giraud, 2008; Glais et al., 2014), or mobile hosts or vectors (Archie & Ezenwa, 2011; Falk & Perkins, 2013; Pereira et al., 2013) is extensive.

### 4.3 Human‐mediated gene flow and consequences for resistant varieties management

Field populations of *H. schachtii* have a similar substructuring patterns than wild populations, suggesting that active dispersal capabilities of nematodes are equivalent in wild and field conditions (Montarry et al., 2015). By contrast, in agrosystems, low genetic differentiation suggests that nematode populations are connected through gene flow among fields separated by distances that reach 150 km. Indeed, maximum pairwise *F*_ST_ reached .1 (Plantard & Porte, 2004; Porte et al., 1999), which is three times less than what we observed across a similar geographical scale in wild populations. These studies attributed the scale of gene flow in agrosystems to soil transport during sugar beet crops harvesting which is characterized by the loss of several tons of soil per hectare and per harvest (Ruysschaert, Poesen, Wauters, Govers, & Verstraeten, 2007). The inadvertent transport of a massive quantity of soil may passively disperse large numbers of cysts, resulting in higher levels of gene flow among cultivated fields than among wild *H. schachtii* populations. Anthropogenic influence on pathogen dispersal has already been reported for several species (Lebarbenchon et al., 2008; Morgan et al., 2012), including nematodes. Patterns of population genetic structure matched those of agricultural practices within and among field populations of *G. tabacum* and *X. index* (Alenda et al., 2014; Villate et al., 2010), and potato tuber trade favors the spreading of *G. pallida* and *G. rostochiensis* worldwide (Boucher et al., 2013; Plantard et al., 2008).

Passive dispersal of cysts in fields results in a higher dispersal capability of nematodes than of their crop hosts. In agrosystems, gene flow is thus a source of genetic variation that facilitates the adaptation of pathogen populations to their local hosts (Gandon et al., 1996; Greischar & Koskella, 2007; Morgan et al., 2005; Thrall & Burdon, 2002). This phenomenon can be strengthened by agrosystems characteristics, such as large‐scale host uniformity (Montarry, Glais, Corbiere, & Andrivon, 2008). Human‐mediated gene flow among fields may thus reduce the durability of resistant varieties used to control crop pathogens.

Limiting accidental gene flow among parasite populations may thus be a worthwhile improvement of the management of field nematode populations because it (i) would isolate avirulent from virulent populations and (ii) reduce the effective population size of field nematode populations. The influence of genetic drift would be favored within populations and may lighten the selective pressure imposed by sugar beet cultivars and thus decrease the ability of nematode populations to overcome resistance over large spatial scales. Such confinement strategies have already been strongly advocated by several authors (Burdon & Thrall, 2008; Stukenbrock & McDonald, 2008) but represent a real challenge. Indeed, they would require a rigorous monitoring of all fields belonging to the same farm to detect infested ones and to prevent soil mixing among fields by, for example, cleaning agricultural equipment. Similarly, in case of machine sharing among agricultural exploitations, a tight collaboration between farmers of the same production area would be helpful to manage parasite dissemination at higher spatio‐temporal scales. These approaches have already been proposed for aerial fungi and would be suitable to maintain the whole pathogen metapopulation maladapted to its hosts and to prevent epidemics (Bousset, 2014; Bousset & Chèvre, 2013). However, all these examples are mostly time‐consuming for farmers which may make them difficult to implement in practice, or they still have to be developed. Thus, additional experimentations and developments will be necessary to fully integrate our conclusions in sugar beet production systems.

## 5 Conclusion

This study used temporal sampling to investigate the genetic structure of wild populations of the beet cyst nematode. Wild populations of *H. schachtii* were characterized by a nonequilibrium population structure, weak levels of gene flow beyond the scale of the host plant, and a non‐negligible impact of genetic drift. This pattern appeared stable over a short period of time, suggesting no isolated disturbance. A wild population of *H. schachtii* appears thus to be defined under the host plant scale, which suggests that human activities strongly influence passive dispersal among field nematode populations. Thus, the management of durable crops’ genetic protection against telluric pathogens may gain from developing methods that combine passive dispersal limitation with other methods that help reduce parasite effective population sizes. Finally, this study illustrates how worthy would be data on the genetic structure of wild and field populations of other plant‐parasitic nematodes species which are currently still lacking.

## Data Archiving Statement

Data for this study are available from the Dryad Digital Repository: http://dx.doi.org/10.5061/dryad.8g5n6.
