# Supplementary material for: Temporal sampling helps unravel the genetic structure of naturally occurring populations of a phytoparasitic nematode. 2. Separating the relative effects of gene flow and genetic drift
Source: Evol Appl. 2016 Jul 22;9(8):1005–16. doi: 10.1111/eva.12401 (PMC4999530; doi:10.1111/eva.12401)

Figure S1: Spatial distribution of sampled sea beet host plants in the four surveyed beaches. The black dots correspond to the plants selected for nematodes genotyping (see text).

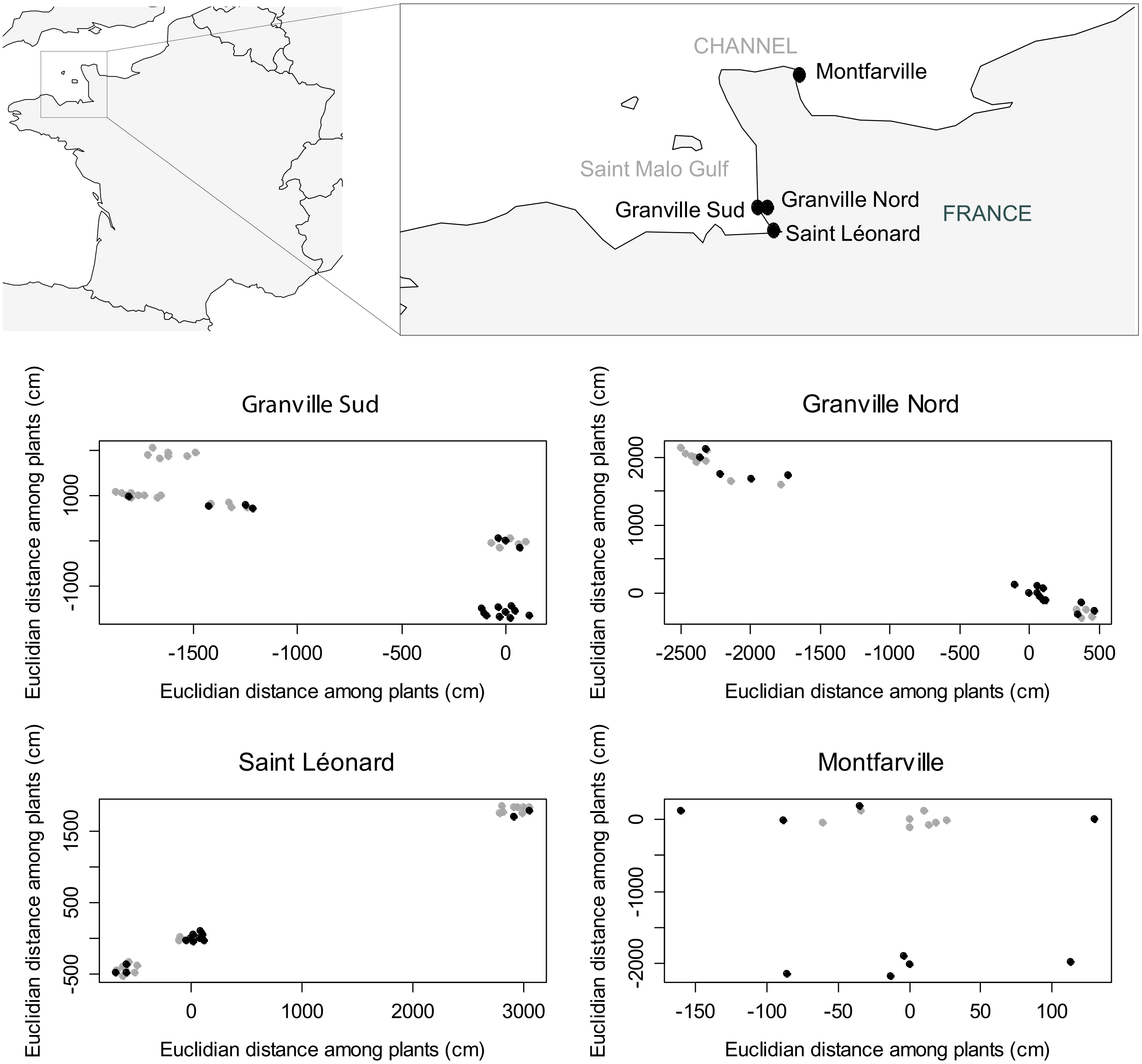

Supplement: Supplementary file 1 [file EVA-9-1005-s001.pdf]
