# Supplementary material for: Temporal sampling helps unravel the genetic structure of naturally occurring populations of a phytoparasitic nematode. 2. Separating the relative effects of gene flow and genetic drift
Source: Evol Appl. 2016 Jul 22;9(8):1005–16. doi: 10.1111/eva.12401 (PMC4999530; doi:10.1111/eva.12401)

Figure S2: Spatial distribution of genetic diversity within each surveyed beach. A, B, E, F, I, J, M, N: synthetic map of the two first local scores of sPCA. Each nematode population was mapped by colour coding its two sPCA lagged scores as intensity of a given colour channel as described in Menozzi et al. (1978). C, D, G, H, K, L, O, P: Spatial autocorrelograms describing the variation of pairwise kinship coefficients Fij among nematode individuals over spatial distance classes. Dotted lines represent 95% confidence interval and error bar standard errors. Black points correspond to values significantly departing from 0 and grey points correspond to non-significant values.

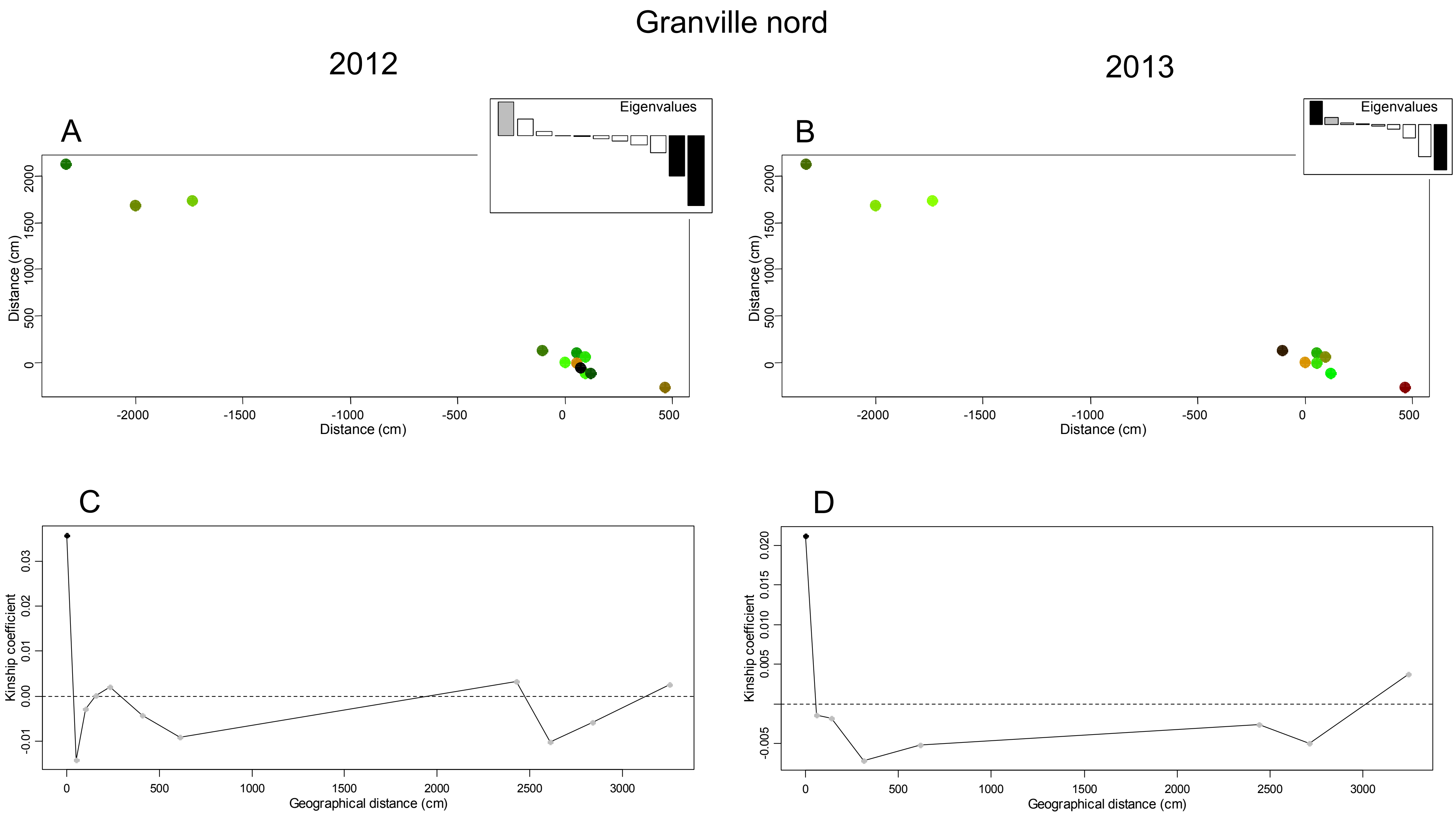

Figure S2, continued

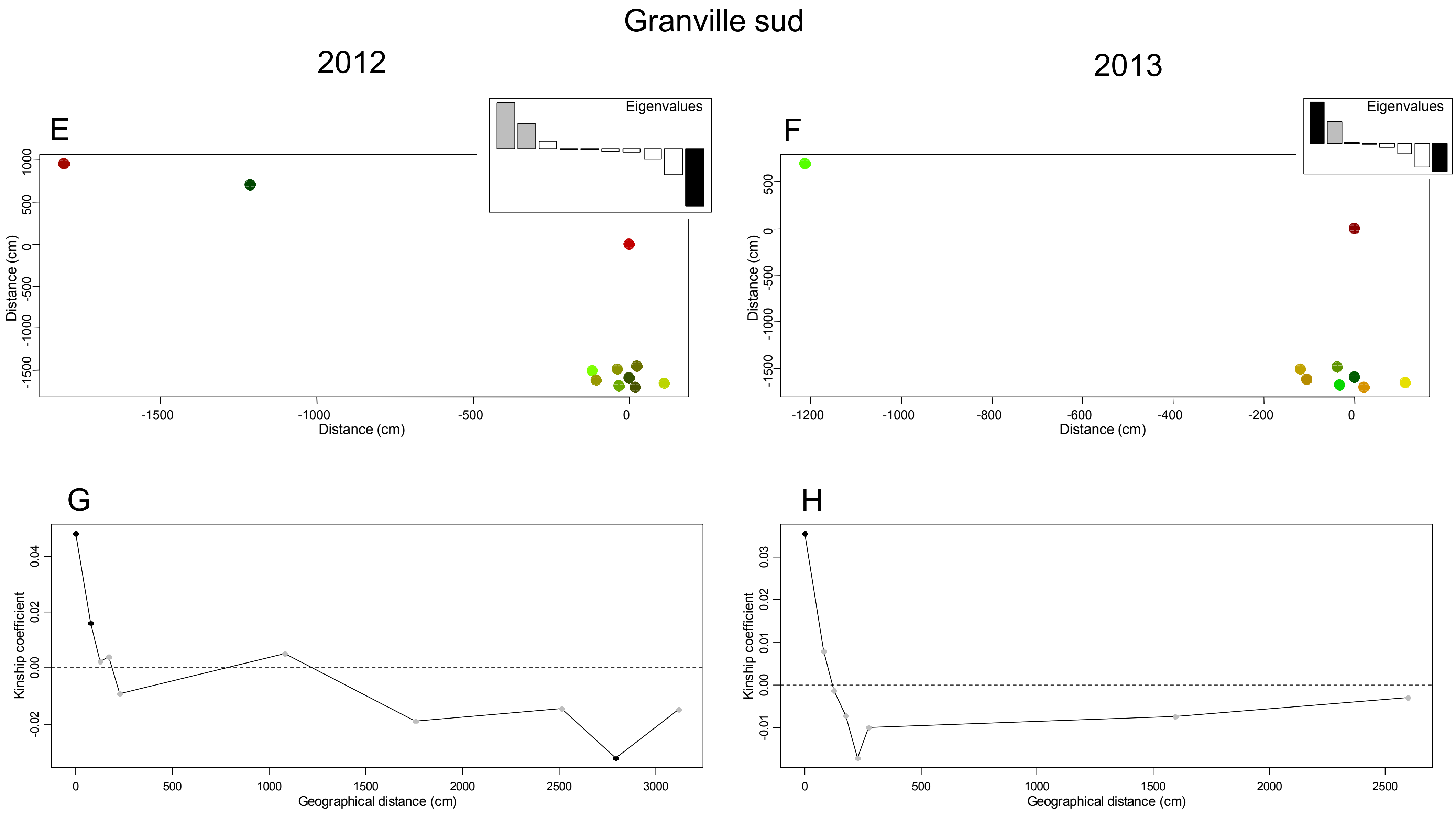

Figure S2, continued

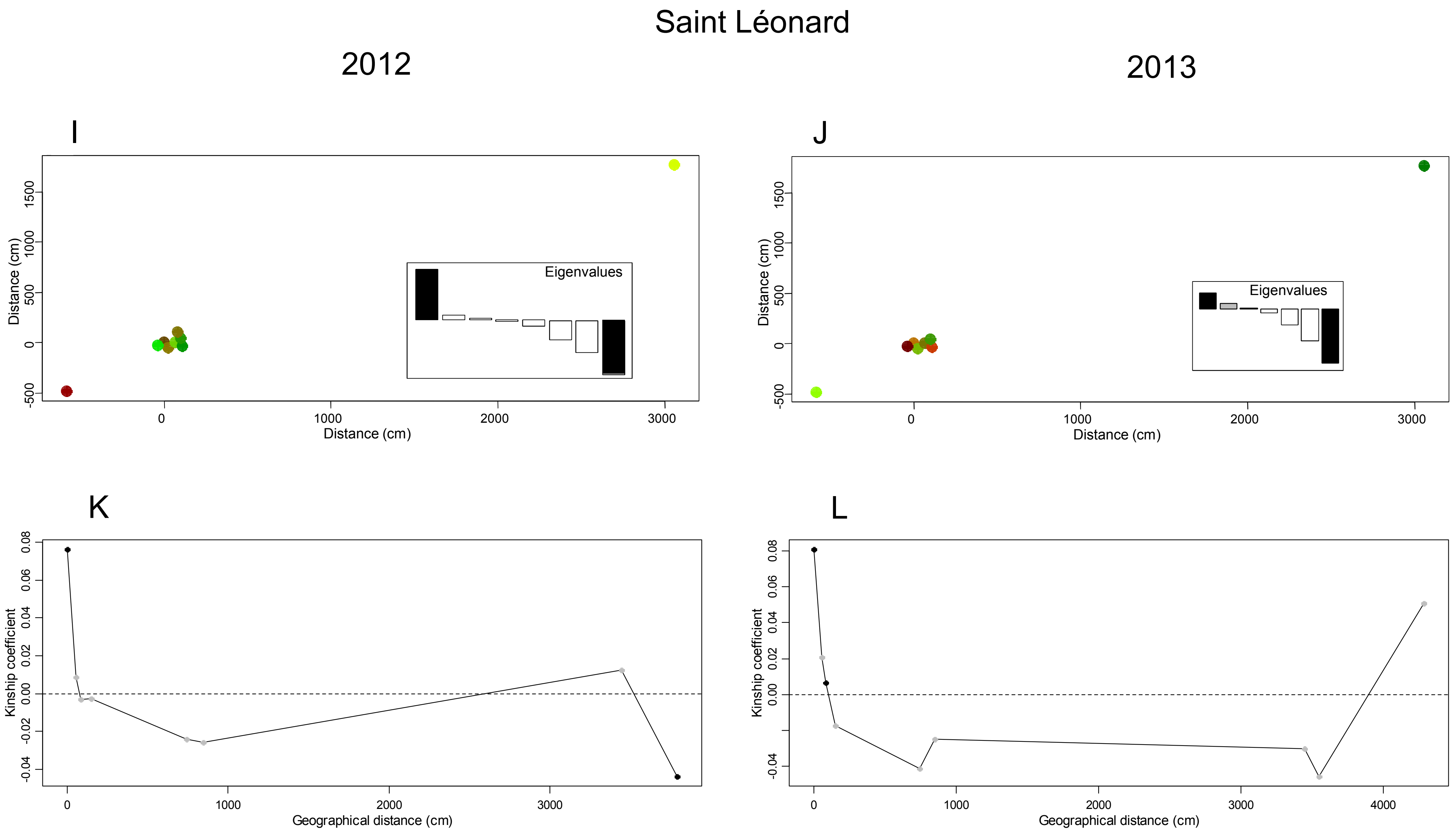

Figure S2, continued

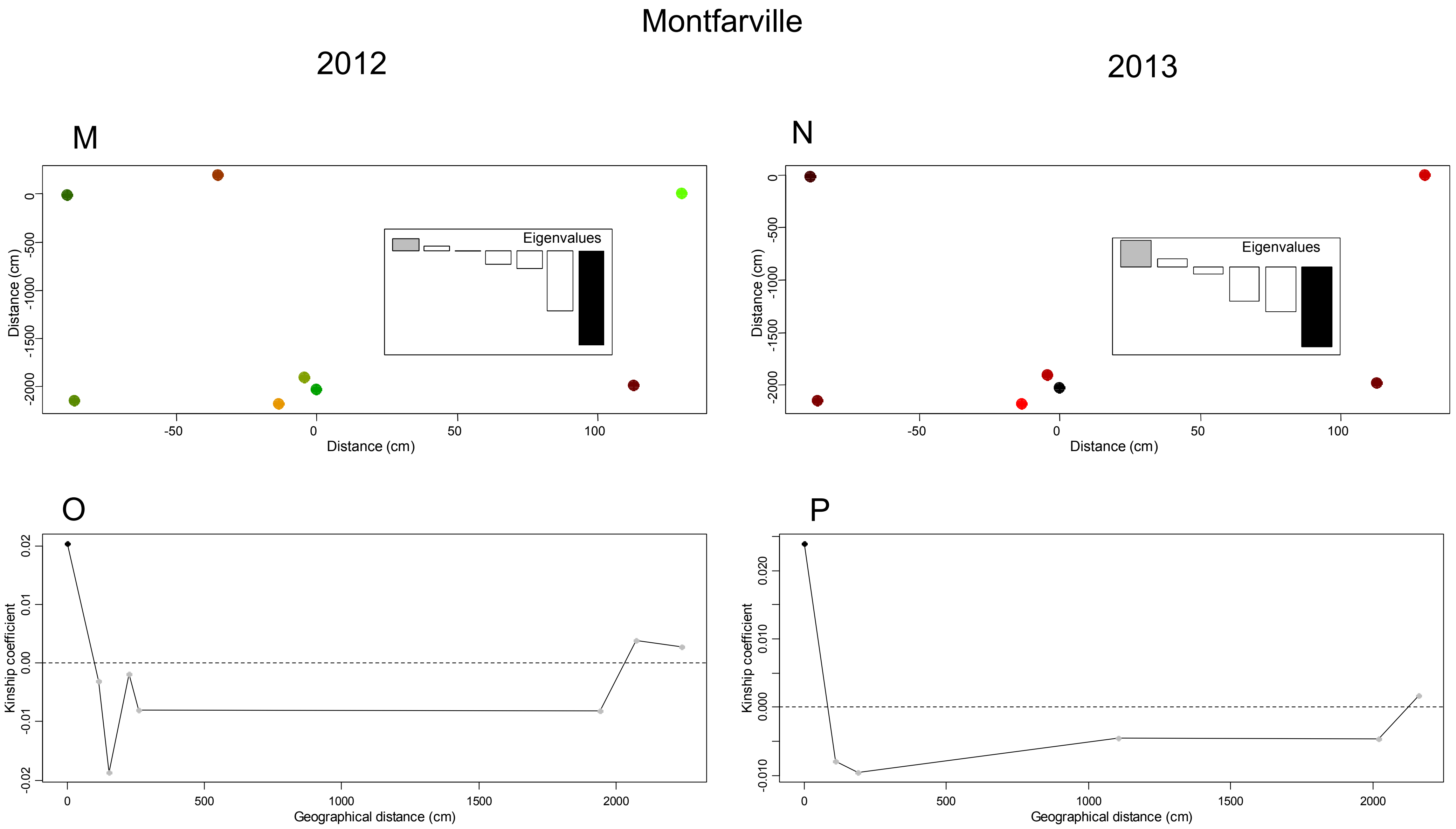

Supplement: Supplementary file 2 [file EVA-9-1005-s002.pdf]
