# Supplementary material for: Temporal sampling helps unravel the genetic structure of naturally occurring populations of a phytoparasitic nematode. 2. Separating the relative effects of gene flow and genetic drift
Source: Evol Appl. 2016 Jul 22;9(8):1005–16. doi: 10.1111/eva.12401 (PMC4999530; doi:10.1111/eva.12401)

Figure S3: Temporal change in the mean (A) and variance (B) in pairwise FST values under a pure drift process. Dark and light grey shapes represent the 95% confidence interval of simulated values obtained after 4 and 10 generations respectively. Dotted lines indicate mean and variance of FST in 2013. Ne: effective population size. The x-axis is on a log scale.

A

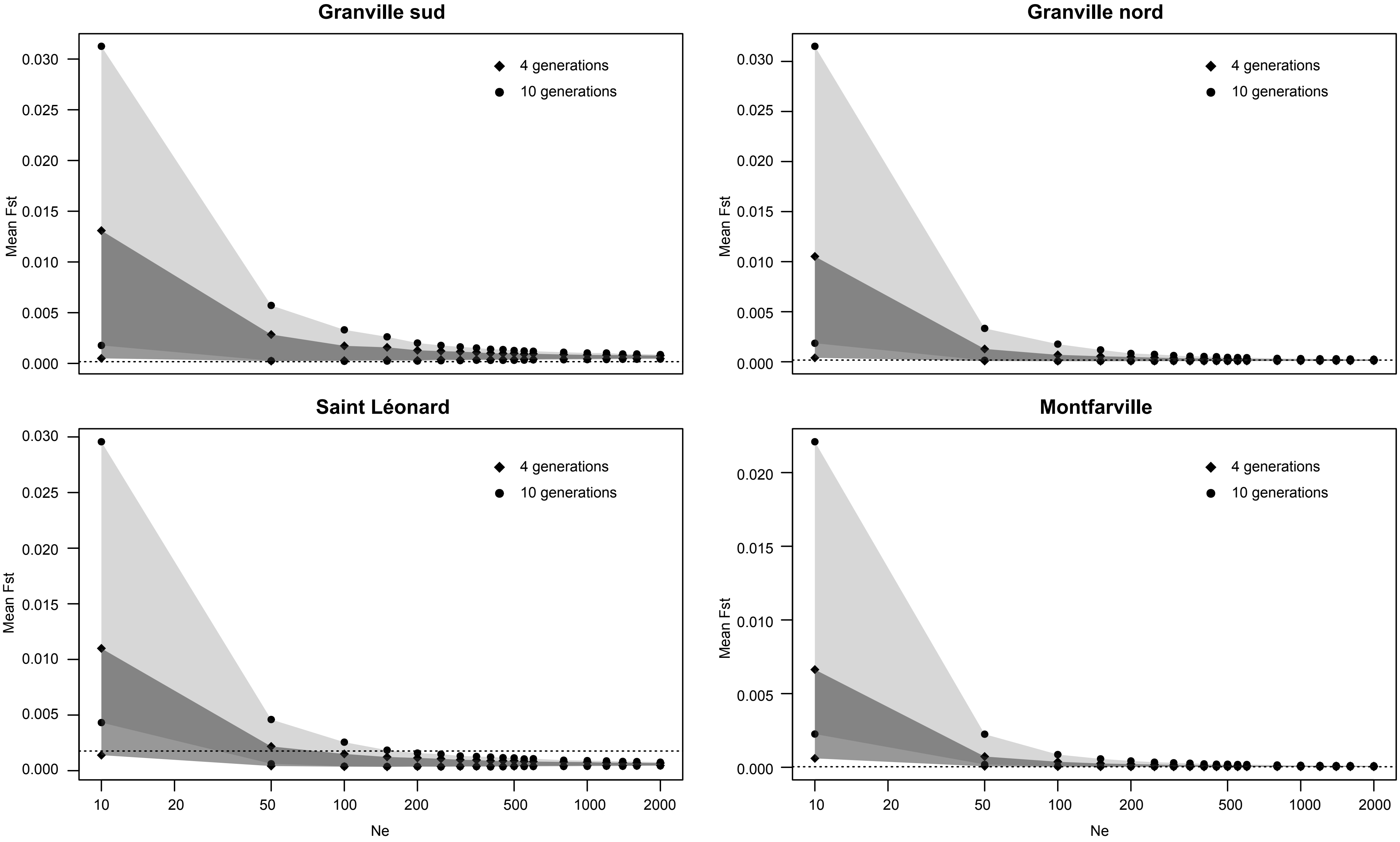

Figure S3, continued

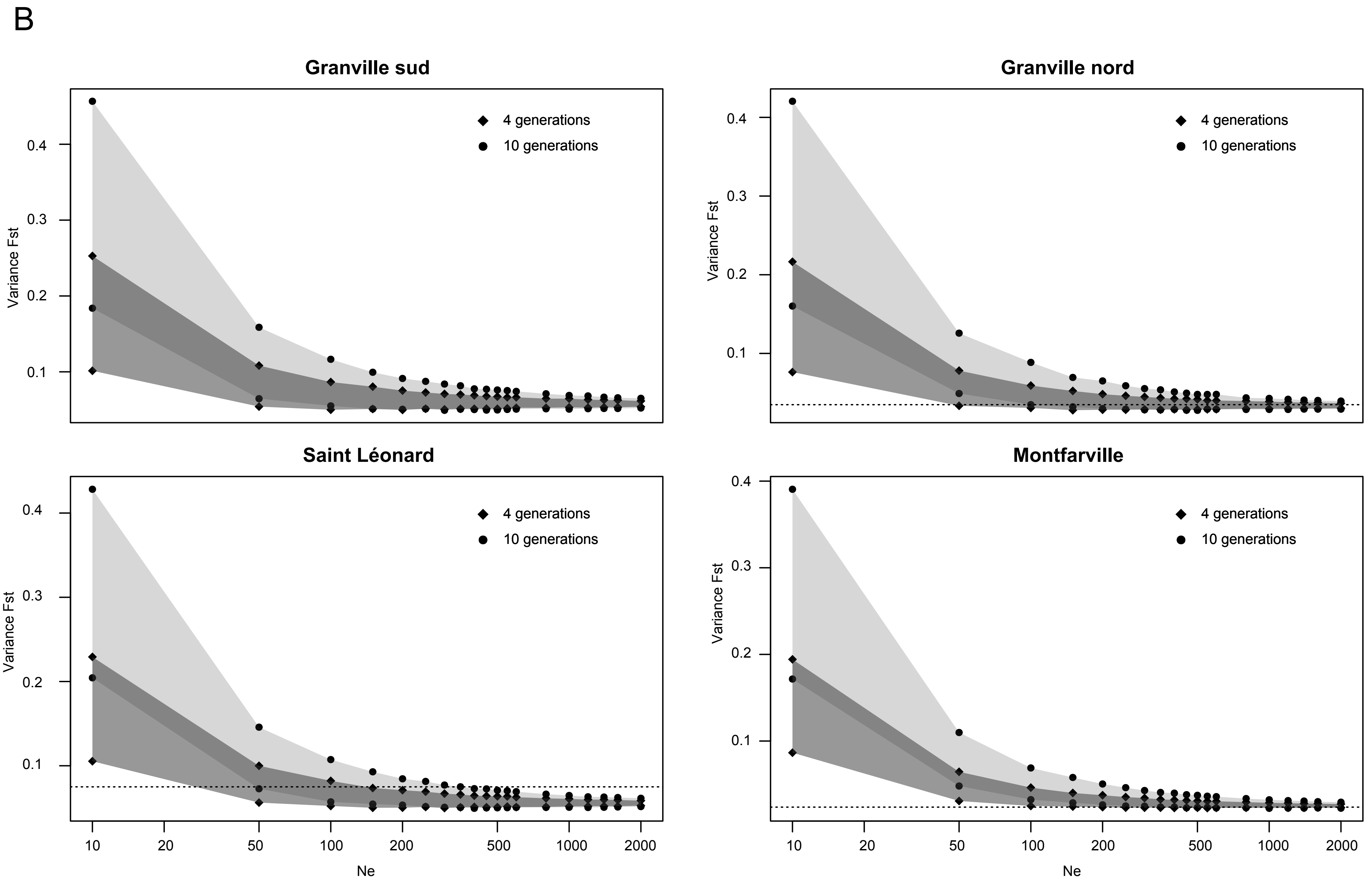

Supplement: Supplementary file 3 [file EVA-9-1005-s003.pdf]
